# Nursing care activities based on documentation

**DOI:** 10.1186/s12912-019-0352-0

**Published:** 2019-08-16

**Authors:** Mira Asmirajanti, Achir Yani S. Hamid, Rr. Tutik Sri Hariyati

**Affiliations:** 1 Nursing Program, Faculty of Health Sciences, Esa Unggul University, Jakarta, 11510 Indonesia; 2 Faculty of Nursing Universitas Indonesia, Jln. Prof. Dr. Bahder Djohan, Kampus UI, Depok, West Java 16424 Indonesia

**Keywords:** Nursing activity, Nursing documentation, Quality of nursing

## Abstract

**Background:**

Nurses engage in various activities from the time of a patient’s admission to his or her discharge from the hospital, helping patients to meet their needs. Each of the activities should be documented properly as authentic and crucial evidence. This study aimed to identify nursing activities in the delivery of nursing care based on the documentation completed.

**Methods:**

A quantitative design with a retrospective approach was used, in which 240 medical records from Dr. Kariadi Hospital in Semarang, dating from July through September 2016, were obtained and assessed. The records were randomly selected based on the 10 most common medical and surgical diseases and a hospital stay of more than 3 days. The instrument for collecting the data from the patient progress notes used an observations form. The data were analyzed using univariate statistics and needed to be at least 80% of the values for a certain criteria for it to be considered. The results were analyzed to compare the standard of care.

**Results:**

It was revealed that nursing activities in the delivery of nursing care were insufficient. These activities, according the standard of nursing activities, included the assessment of the functional status of decubitus risk (20.8%), biological status (0.4%), formulation of a nursing diagnosis (20.8%), identification of patients’ home needs (41.3%), quality of life (66.3%), collaboration intervention in drug administration (60.8%), monitoring of vital signs (23.3%), monitoring of daily living activities (37.5%), mobilization/rehabilitation (37.5%), outcome (46.7%), and resume activities nursing (0.8%).

**Conclusions:**

Nursing activities are very important within the hospital and must solve the problems that the patient needs. Every nursing activity should produce documentation with critical thinking. If nursing documents are not clear and accurate, inter-professional communication and an evaluation of nursing care cannot be optimal. Nursing activity and documentation should be continuously directed, controlled, and evaluated by a nurse manager. The quality of nursing activities should always be good to increase patient satisfaction, patient safety, and cost-effectiveness.

## Background

Nurses are involved in many activities in a hospital from patient admission through discharge. They provide continuous 24-h patient care, which is divided into several shifts [1]. Patient care includes performing assessments, stating nursing diagnoses, developing intervention plans, implementing care, and making evaluations to modify or terminate care [2]. Examples of nursing interventions include discharge planning and education, the provision of emotional support, self-hygiene and oral care, monitoring fluid intake and output, ambulation, the provision of meals, and surveillance of a patient’s general condition [3]. The delivery of nursing care should involve the patient. A nurse respectfully communicates, coordinates, and integrates nursing care, provides education and information, and considers the comprehensive and continuous physical and emotional comfort of the patient [4]. In addition, a nurse employs an appropriate strategy to establish a good rapport with a patient and is able to understand a patient’s condition in such a way that they can motivate him or her to actively participate in every nursing activity [5].

Each nursing activity should consider patient safety. Nurses are responsible for preventing patients from falling and from developing pressure ulcers, urinary tract infections, and nosocomial infections [6]. They provide education and information regarding the procedures involved in nursing interventions beforehand and involve patients for their own safety; effective communication is the key to patient safety [7].

Nursing activity that has been completed or that will take place should be properly documented. Accurate documentation and reports play a pivotal role in health services [8]. This documentation is necessary to identify nursing interventions that have been provided to patients and to show patient progress during hospitalization [9]. It is also an indicator of nurse performance and the nursing service quality in a hospital. Documentation provides details of patient condition, nursing interventions that have been provided, and patient response to the intervention(s) [10].

Nursing documentation also serves as an effective tool of inter-professional communication between nurses and other health professionals for delivering ongoing nursing care, evaluating patient progress and outcomes, and providing constant patient protection [11]. High-quality nursing documentation may improve the effectiveness of communication between health professionals in first- and higher-level healthcare facilities [12].

The documentation should be saved for an appropriate length of time and should be concise and clear; complete, accurate, and up-to-date documentation will protect a nurse in a court of law [13]. Correct documentation may encourage a nurse to establish continuity between the diagnosis, intervention, progress, and evaluation of the outcome [14]. A previous study revealed that 54.7% of nursing documents were of poor quality and 71.6% were incomplete [15]. Supervision by the head nurse is required for complete, concise, and accurate documentation of nursing care [16]. The information above provides a platform for managers and nurses to better understand the delivery of nursing care.

## Methods

### Design

A quantitative, cross-sectional, and retrospective study used the medical records of discharged patients. The medical records concerned patients who had been hospitalized for more than 3 days at the medical surgical ward.

### Setting and sample

The study was conducted in DK Hospital of Semarang from October until December 2016. Data were obtained from July to September 2016 from 240 medical records of patients with the 10 most common medical surgical diseases. The 240 medical records were randomly selected by simple random methods based on even and odd numbers. Ethical clearance procedures were followed. Medical records data were maintained confidentially, were used only for research purposes, and were not disseminated for other purposes.

### Data collection

The authors recorded all nursing activities performed by nurses from the time of a patient’s admission until his or her discharge via an observation form that had been developed by referring to patient progress notes. This observation form consists of nursing activities and had been tested for validity and reliability to achieve optimal data. The validity and reliability results were r Alpha > 0.90 and coefficient kappa > 0.80.

### Data analysis

The collected data were assigned codes, inputted into a computer, and cleared of unnecessary information. The data were checked during entry and compilation before analysis. After checking the data for completeness, missing values, and coding questionnaires, data were entered into the computer and analyzed. Univariate analysis was used to identify the frequency and percentage of nursing activities performed. The results were analyzed to compare the standard of care with the hospital accreditation standard and needed to be at least 80% of the values for a certain criteria for it to be considered.

## Results

A total of 240 medical records for patients who had been hospitalized for more than 3 days in the medical surgical ward were obtained and analyzed. Data were obtained from the documentation completed by nurses while providing nursing care for each patient. These activities involved patient identification, assessment, nursing diagnosis formulation, discharge planning, education, intervention, monitoring and evaluation, mobilization/rehabilitation, and nursing outcomes. The results are presented in Table 1 below.

**Table 1:** Description of nursing activities in providing nursing care in Dr. Kariadi Hospital of Semarang (*n* = 240)

Category	Frequency (%)
A Patient Identification	
Name	240 (100%)
Medical record number	240 (100%)
Age	240 (100%)
Weight	232 (96.7%)
Height	232 (96.7%)
B Initial Assessment	
General appearance	240 (100%)
Consciousness level	239 (99.6%)
Vital signs	238 (99.2%)
History of allergy	240 (100%)
Nutritional screening	240 (100%)
Pain	238 (99.2%)
C Functional Status	
Barthel index	238 (99.2%)
Risk of fall	239 (99.6%)
Risk of pressure ulcer	50 (20.8%)
Educational need	231 (96.3%)
Cultural need	211 (87.9%)
D Focused Assessment	
Biology	1 (0.4%)
Psychology	240 (100%)
Spiritual	235 (97.9%)
Cultural	212 (88.3%)
E Nursing Diagnosis	50 (20.8%)
F Discharge Planning	
Identification during hospitalization	239 (99.6%)
Identification of requirements at home	99 (41.3%)
Quality of life	159 (66.3%)
G Education	237 (98.8%)
H Intervention	
Vital signs intervention	213 (88.8%)
Other interventions	238 (99.2%)
Collaboration: Drug administration	146 (60.8%)
I Monitoring and Evaluation	
Monitoring vital signs	56 (23.3%)
Monitoring activity of daily living	91 (37.9%)
Other monitoring	239 (99.6%)
J Mobilization/Rehabilitation	90 (37.5%)
K Outcome	112 (46.7%)
L Discharge Planning/Education	
Nursing resumé	2 (0.8%)
Initial of nurse in charge	239 (99.6%)

The results show that the nurses performance on some nursing activities were below standard (80%). Some nursing activities which needed to be optimized including the assessment of functional status, risk of a pressure ulcer (20.8%), assessment of biological aspect (0.4%), formulation of a nursing diagnosis (20.8%), collaboration in drug administration (60.8%), monitoring of vital signs (23.3%), monitoring of activities of daily living (ADL) (37.5%), mobilization/rehabilitation (37.5%), nursing outcome (46.7%), identification of patients’ home (41.3%), quality of life (66.3%), and nursing activities resumé (0.8%).

The results also indicated that nursing activities were not implemented in compliance with the nursing process; for example, some nurses had not properly performed a biological assessment before proceeding to formulate their diagnosis and perform an intervention. Although the interventions were properly executed, the mobilization and monitoring activities could be improved. Nurses rarely formulated a nursing diagnosis before the expected outcome; however, these two activities should be performed in order, since it may affect the planned nursing intervention. The nurses did not properly identify the patients’ home needs in discharge planning, nor did they create an optimal nursing activities resumé.

## Discussion

The results revealed that nursing activities to solve problems and meet patient needs in the provision of nursing care were not systematically performed and critical thinking was not applied during the nursing process. A previous study asserted that the nursing process incorporates the assessment, nursing diagnosis, planning, implementation, evaluation, and documentation [16]. The phases in the nursing process are interconnected and become a continuous cycle. Therefore, steps in this process are interrelated, interactive, and cannot stand alone [17].

It was also shown that some nurses did not perform a biological assessment, yet they proceeded to formulate nursing diagnoses and perform interventions. A nursing diagnosis, however, should be based on the assessment result and used as reference in determining the intervention [18]. Nurses should consider using a nursing process that complies with the input, process, and output in formulating an intervention, since it may affect the quality of care and patient safety in general [19]. Patient safety is a fundamental concern for all nurses and health professionals, from the patient’s admission to the hospital until discharge; therefore, it is required that every nursing process is implemented according to the standards applied and in a sustainable manner. If these standards are not observed, then the nurses and other health professionals would not meet patient needs and may even compromise patient safety.

It was shown that nursing activities in identifying the patients’ home needs and quality of life during discharge planning were not properly implemented. Discharge planning is a crucial nursing activity that facilitates a patient’s readiness regarding his or her discharge from the hospital; it allows a patient to be safely transferred from the hospital to their own home. Lack of nursing support in this activity has previously resulted in an increased number of patient readmissions [20]. Although discharge planning also involves other healthcare professionals, the nurse has the longest amount of time to interact with the patient. The nurse should understand the patient’s condition, recognize their ability to accept it, and improve the readiness of the patient and their family for continuing care at home.

The collaboration intervention of drug administration was not fully implemented. Nurses should provide education regarding the function, composition, and side effects of a drug and adverse reactions that may occur with uncontrolled use. Therefore, a nurse should ensure that a patient has been properly informed of the drug prescribed by a physician. A previous study revealed that collaboration in drug administration in provision of nursing care may improve patient satisfaction and reduce their stress and anxiety [5].

The findings revealed that nursing activities in vital signs and ADL monitoring were not correctly implemented. Monitoring is a critical nursing activity and identifies a patient’s condition and ability to meet their daily needs so that a nurse may devise an appropriate intervention. A previous study revealed that nurses played a pivotal role in helping patients to recuperate by performing an assessment, monitoring, intervention, evaluation, and provision of support [21], immediately recognizing a change in a patient’s condition, health promotion, preventing morbidity, improving patient satisfaction, and quality of care.

In the present study, nursing activities in patient mobilization/rehabilitation were not properly executed. Patient mobilization/rehabilitation is an activity that must be implemented immediately after a patient’s hemodynamic parameters are stabilized in order to improve their physical condition. A previous study stated that nurses should pay heed and motivate patients in rehabilitation to ensure effective and cost-effective care [22].

The present findings also showed that nursing activities in deciding the patient outcome were not optimal. The determination of outcome serves to evaluate how much progress has been made by a patient following the delivery of nursing care. Indeed, one study claimed that the determination of outcome reflected the unique contribution of nursing care toward patient safety [23].

The present findings of improper nursing activities may have resulted from numerous factors, such as having to perform a large number of non-nursing duties, manual documentation, a lack of standards in documenting patient progress notes, and the exclusion of nursing care in calculating remuneration.

All nursing activities should be properly documented as authentic information and used to evaluate nursing care and professional competency. Nursing documentation is an essential component of professional practice to improve the quality of nursing care and should be accurate and complete [24, 25]. Complete documentation encourages nurses to work effectively and appropriately [14].

## Conclusion

Some nursing activities have been done properly, but they were not continuously in compliance with the nursing process. Nursing care was not systematically performed and critical thinking was not applied during the nursing process. Many nurses did not do a biological assessment, yet they proceeded to formulate nursing diagnoses and perform interventions. Nursing activities in identifying patients’ home needs and quality of life during discharge planning, collaboration intervention of drug administration, vital signs and ADL monitoring, patient mobilization/rehabilitation. and deciding the patient outcome were not properly implemented.

The nursing process should be properly implemented in order to improve patient and nurse satisfaction, quality of care, patient safety, and cost-effectiveness, as well as to reduce the average length of stay. A nurse who has completed nursing activities is required to document the care provided, according to the standard applied. Nursing activities and documentation may be more likely to be optimal if they are regularly directed, controlled, and evaluated by the nurse manager. A nurse and patient satisfaction survey should also be periodically conducted to evaluate the quality of nursing activities in the delivery of nursing care for patients.

## Abbreviation

ADL: Activities of daily living
